# Author Correction: Fast-moving stars around an intermediate-mass black hole in ω Centauri

**DOI:** 10.1038/s41586-024-08017-4

**Published:** 2024-09-17

**Authors:** Maximilian Häberle, Nadine Neumayer, Anil Seth, Andrea Bellini, Mattia Libralato, Holger Baumgardt, Matthew Whitaker, Antoine Dumont, Mayte Alfaro-Cuello, Jay Anderson, Callie Clontz, Nikolay Kacharov, Sebastian Kamann, Anja Feldmeier-Krause, Antonino Milone, Maria Selina Nitschai, Renuka Pechetti, Glenn van de Ven

**Affiliations:** 1 Max Planck Institute for Astronomy, Heidelberg, Germany; 2 Department of Physics and Astronomy, University of Utah, Salt Lake City, UT USA; 3 Space Telescope Science Institute, Baltimore, MD USA; 4 AURA for the European Space Agency (ESA), Space Telescope Science Institute, Baltimore, MD USA; 5 INAF, Osservatorio Astronomico di Padova, Padova, Italy; 6 School of Mathematics and Physics, The University of Queensland, St Lucia, Queensland Australia; 7 Facultad de Ingeniería y Arquitectura, Universidad Central de Chile, La Serena, Chile; 8 Leibniz Institute for Astrophysics, Potsdam, Germany; 9 Astrophysics Research Institute, Liverpool John Moores University, Liverpool, United Kingdom; 10 Department of Astrophysics, University of Vienna, Wien, Austria; 11 Dipartimento di Fisica e Astronomia ‘Galileo Galilei’, Università Degli Studi di Padova, Padova, Italy

**Keywords:** Compact astrophysical objects, Galaxies and clusters, Stars

Correction to: *Nature* 10.1038/s41586-024-07511-z Published online 10 July 2024

In the version of the article initially published, the third equation in the “The escape velocity provides a minimum black hole mass” section was presented incorrectly; the original and revised equations are shown below. The error was in description, only, and does not impact the results. Also, an error in conversion of coordinates from relative to absolute coordinates led to a small offset in the reported right ascension coordinate for the minimum mass black hole location and the MCMC center. In the Methods sentence now reading “If we assume that all five robustly detected stars are bound to the black hole, the lower limit is about 8,200*M*_☉_ and the minimum mass location is only 0.3″ away from the AvdM10 (ref. 6) centre at the location RA = 201.6966908° and Dec = −47.4795066°,” the coordinate “201.6966908°” replaces “201.6967370°,” while in the Methods text now reading “The coordinates of the MCMC based centre estimate are RA = 201.6970988° and Dec = −47.4794533°,” the coordinate “201.6970988°” replaces “201.6970128°.” The equation and text are updated in the HTML and PDF versions of the article.

**Original equation**

1 $${M}_{\text{BH}} > \sqrt{{\left(\frac{{v}_{3\text{D}}^{2}{r}_{3\text{D}}}{2G}\right)}^{2}-{v}_{\text{esc., cluster}}^{2}}\ge \sqrt{{\left(\frac{{v}_{2\text{D}}^{2}{r}_{2\text{D}}}{2G}\right)}^{2}-{v}_{\text{esc., cluster}}^{2}}$$

**Revised equation**

2 $${M}_{\text{BH}} > \frac{\left({v}_{3\text{D}}^{2}-{v}_{\text{esc., cluster}}^{2}\right){r}_{3\text{D}}}{2G}\ge \frac{\left({v}_{2\text{D}}^{2}-{v}_{\text{esc., cluster}}^{2}\right){r}_{2\text{D}}}{2G}$$

